# Identification and Characterization of NAC Transcription Factors Involved in Pine Wilt Nematode Resistance in *Pinus massoniana*

**DOI:** 10.3390/plants14152399

**Published:** 2025-08-03

**Authors:** Zhengping Zhao, Jieyun Lei, Min Zhang, Jiale Li, Chungeng Pi, Jinxiu Yu, Xuewu Yan, Kun Luo, Yonggang Xia

**Affiliations:** 1 Hunan Academy of Forestry, Changsha 410004, China; 2 College of Plant Protection, Hunan Agricultural University, Changsha 410125, China; ljieyunjy@163.com (J.L.);; 3 Youxian County Forestry Bureau, Zhuzhou 412300, China

**Keywords:** NAC family, *Bursaphelenchus xylophilus*, pine wilt disease, transcription factor, subcellular localization, hormones

## Abstract

*Pinus massoniana* Lamb. is an economically important conifer native to China. However, it is highly susceptible to the pine wood nematode (*Bursaphelenchus xylophilus*, PWN), the causal agent of pine wilt disease (PWD), resulting in substantial ecological and economic losses. To elucidate potential molecular defense mechanisms, 50 NAC (NAM, ATAF1/2, and CUC2) transcription factors (PmNACs) were identified in the *P. massoniana* genome. Phylogenetic analysis divided these PmNACs into seven subfamilies, and motif analysis identified ten conserved motifs associated with stress responses. Twenty-three genes were selected for expression analysis in various tissues and under exogenous salicylic acid (SA), methyl jasmonate (MeJA), and PWN infection. Six genes (*PmNAC1*, *PmNAC8*, *PmNAC9*, *PmNAC17*, *PmNAC18*, and *PmNAC20*) were significantly up-regulated by both hormonal treatment and PWN infection, implying their involvement in JA/SA-mediated immune pathways. Functional characterization showed *PmNAC8* is a nuclear-localized transcription factor with autoactivation activity. Furthermore, transient overexpression of *PmNAC8* in *Nicotiana benthamiana* induced reactive oxygen species (ROS) accumulation and necrotic lesions. Collectively, these results elucidate NAC-mediated defense responses to PWN infection in *P. massoniana* and identify candidate genes for developing PWD-resistant pine varieties.

## 1. Introduction

*Pinus massoniana* is the most widely distributed and abundant pine species in China and possesses considerable ecological and economic importance. This species exhibits extensive utilization of all plant parts, as its pollen, roots, needles, and cones are used for medicinal purposes in the pharmaceutical and nutraceutical industries [1,2]. Moreover, *P. massoniana* plays a pivotal role in controlling soil erosion and promoting carbon sequestration in degraded forest soils [3]. Its upright growth habit makes it particularly suitable for ecological reforestation and landscape greening, thereby supporting sustainable forestry development in southern China [4].

The spread of the quarantine pest *Bursaphelenchus xylophilus* (pine wood nematode, PWN) causes extensive outbreaks of pine wilt disease (PWD) in coniferous trees, with native *P*. *massoniana* populations suffering particularly severe damage due to their high susceptibility [5]. PWN primarily invades host trees through wounds created by insect vectors, such as *Monochamus alternatus* [6], rapidly proliferates within resin canals, and systematically disrupts host physiological functions [7]. During infection, parenchyma cells excessively synthesize terpenoids; however, the volatilization of these terpenoids leads to xylem cavitation. Simultaneously, PWN feeding within stems impedes water conduction [8,9]. These mechanisms result in extensive mortality of *Pinus* species, including *P. massoniana* and *Pinus koraiensis*, severely threatening forest ecosystem stability, reducing biodiversity, and causing substantial economic losses and high epidemic management costs to the timber industry [10]. Current preventive measures depend mainly on import and export quarantine practices, while primary mitigation strategies consist of environmentally harmful burning and fumigation of infected timber [10]. Although the development of resistant cultivars offers potential, its lengthy breeding cycle hampers practical implementation [11]. Consequently, inadequate control methods, complex epidemic management, and climate-driven expansion of PWN-suitable habitats are intensifying China’s forestry crisis, representing an ongoing threat to ecological security [12].

Plant recognition of pathogens or pests activates pattern-triggered immunity (PTI) or effector-triggered immunity (ETI), inducing the accumulation of defense hormones such as salicylic acid (SA), jasmonic acid (JA), and ethylene (ET) [13]. In *P. massoniana*, JA and SA concentrations exhibit significant elevation following infection by *B*. *xylophilus*, thereby initiating downstream immune responses against nematode invasion [14]. During PWN infection, plants undergo intense oxidative stress. Reactive oxygen species (ROS)—generated through plant defense mechanisms, necrotic cell decomposition, or nematode activity—serve as signaling molecules during early nematode recognition [15]. These oxidative molecules activate defense responses, enhance cell wall reinforcement via cross-linking, and display potential nematotoxic effects [16]. Quantitative analysis in *P. massoniana* demonstrated that both superoxide anion (O_2_^−^) and hydrogen peroxide (H_2_O_2_) surge during early infection (24 h post-inoculation, hpi) in both resistant and susceptible genotypes. Notably, resistant plants exhibited a progressive decline in ROS levels between 15 and 30 days post-inoculation (dpi), whereas susceptible counterparts maintained elevated concentrations [17].

The NAC (NAM, ATAF1/2, and CUC2) transcription factors represent a plant-specific TF family that plays pivotal roles in developmental processes and stress responses [18]. Structurally, NAC proteins possess a highly conserved N-terminal NAC domain, which is further divided into five subdomains (A–E) [19]. This domain facilitates DNA binding and protein interactions, thereby enabling NAC TFs to regulate various biological processes, including but not limited to shoot apical meristem formation, floral organogenesis, lateral bud development, phytohormone signaling, and defense responses [19,20]. At the structural level, the NAC domain exhibits a unique fold that differs from classical helix–turn–helix motifs, consisting of a twisted β-sheet surrounded by helical elements. The formation of functional dimers by this domain offers a structural framework for elucidating NAC protein functions at the molecular level [21]. Furthermore, NAC family members orchestrate gene expression through binding to cis-acting elements located in promoter regions, thus coordinating secondary cell wall biosynthesis, leaf senescence, fruit ripening, and secondary metabolite synthesis [22].

Emerging evidence indicates that NAC transcription factors play a distinctive role in coordinating the crosstalk between biotic and abiotic stress response networks, in contrast to WRKY/MYB transcription factors that typically function within specific stress pathways [19,23]. As central regulators, NAC proteins integrate signaling from SA, JA, and abscisic acid (ABA) to orchestrate systemic acquired resistance [24]. For instance, the NAC protein ATAF1 was shown to suppress nematode proliferation through the JA signaling pathway [25]. However, such mechanistic investigations have been largely confined to gramineous crops, while woody plants have been comparatively overlooked. Specifically, the functional contribution of NAC transcription factors to the defense of *P*. *massoniana* against *B*. *xylophilus* (PWN) infection has not yet been systematically characterized. To fill this knowledge gap, our study combines comparative genomics with evolutionary analysis to elucidate the regulatory functions of the NAC family in conferring PWN resistance in this ecologically crucial conifer.

## 2. Results

### 2.1. Biophysical Properties Analysis of P. massoniana NAC Proteins

We identified 50 *P*. *massoniana* NAC family protein sequences along with their corresponding cDNA sequences (Table S1). Comprehensive physicochemical properties analyses showed that these NAC sequences encoded proteins ranging from 111 (*PmNAC39*) to 695 (*PmNAC22*) amino acids, corresponding to predicted molecular weights of 12.8 kDa (*PmNAC39*) to 77.4 kDa (*PmNAC22*). The theoretical isoelectric points (pI) were distributed between 5.0 (*PmNAC2*) and 9.8 (*PmNAC14*), comprising 24 acidic proteins (pI < 7) and 26 basic proteins (pI > 7). Bioinformatic analyses of protein stability demonstrated that 11 PmNACs (including *PmNAC4)* had instability indices < 40, indicating potential stability, while the remaining proteins showed instability indices > 40, suggesting relative instability. The grand average of hydropathicity (GRAVY) values spanned from −0.969 (*PmNAC15*) to −0.049 (*PmNAC24*), where 44 PmNACs exhibited strongly hydrophilic characteristics (GRAVY < −0.5), whereas 6 members displayed hydrophobic tendencies (GRAVY > −0.5). This hydrophobicity could potentially influence their interactions with hydrophilic molecules and/or nuclear localization. Consistent with their putative transcription factor function, subcellular localization predictions strongly suggested nuclear localization for all 50 sequences. These results support their proposed role in modulating gene expression during key phases of plant development. Collectively, our data indicate that these 50 *P. massoniana* NAC family genes are predominantly nuclear-localized transcription factors that may function across varying pH microenvironments to coordinate regulatory processes in this subcellular compartment.

Comprehensive secondary structure prediction analysis revealed substantial variation in structural components as α-helix content spanned from 4.84% (*PmNAC3*) up to 20.41% (*PmNAC26*), while extended strand content varied between 5.04% (*PmNAC7*) and 25.81% (*PmNAC21*). Notably, β-sheet structures were conspicuously absent in all predicted sequences. Random coil content dominated the structural profiles, accounting for 60.49% (*PmNAC41*) to 86.77% (*PmNAC3*) of total secondary structures. These results demonstrate that the 50 PmNACs are structurally characterized by predominant random coil configurations with α-helices and extended strands as supplementary elements. The conserved structural architecture observed throughout the family implies the evolutionary stability of functional domains and suggests functional redundancy in regulatory mechanisms.

### 2.2. Multiple Sequence Alignment Analysis of the P. massoniana NAC Family Proteins

**Figure 1:**
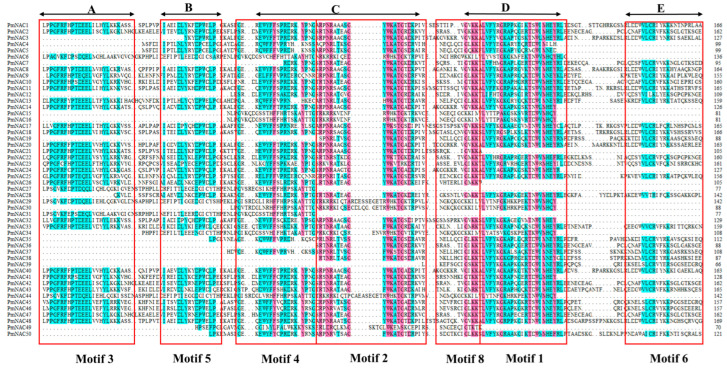
Multi-sequence alignment of conserved domains of Masson pine protein sequences. The red part indicates sequence similarity ≥ 75%, and the blue area indicates sequence similarity ≥ 50%. (**A**–**E**) represent the five canonical structural subdomains of the NAC family. The blank area within the red box indicates that the gene is missing the corresponding fragment. Motif 3 constitutes subdomain A, Motif 5 constitutes subdomain B, Motif 2 and Motif 4 constitute subdomain C, Motif 1 and Motif 8 constitute subdomain D, and Motif 6 constitutes subdomain E.

Multiple sequence alignments of the 50 PmNACs were conducted (Figure 1), which revealed that the majority of sequences contain all five relatively conserved NAC-specific subdomains (A, B, C, D, and E). However, twelve proteins (*PmNAC3*, *PmNAC4*, *PmNAC5*, *PmNAC12*, *PmNAC15*, *PmNAC19*, *PmNAC34*, *PmNAC35*, *PmNAC36*, *PmNAC37*, *PmNAC38*, and *PmNAC39*) were found to lack the A subdomain, and six of these (*PmNAC12*, *PmNAC15*, *PmNAC19*, *PmNAC36*, *PmNAC38*, and *PmNAC39*) also lacked the B subdomain. Furthermore, the C subdomain was identified as incomplete in *PmNAC36* and *PmNAC39*. Additionally, five other protein sequences (*PmNAC21*, *PmNAC26*, *PmNAC27*, *PmNAC32*, and *PmNAC41*) showed absence of the E subdomain together with the downstream C-terminal regulatory region. Taken together, these results strongly suggest that although most PmNACs are predicted to be fully functional, a subset of PmNACs with incomplete subdomains may depend on interaction with other proteins to achieve their biological functions.

### 2.3. Classification and Phylogenetic Analysis of PmNACs

**Figure 2:**
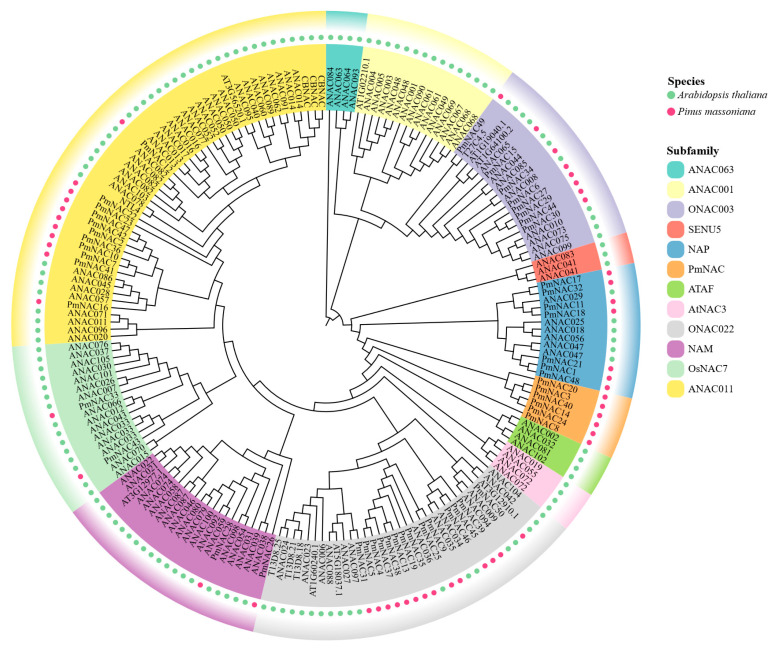
Phylogenetic analysis of NAC genes from *P*. *massoniana* and *A*. *thaliana*. The unrooted phylogenetic tree was constructed using the Maximum Likelihood (ML) method with 1000 bootstrap replicates. NAC proteins from *P. massoniana* and *A. thaliana* are indicated by red and green dots, respectively. The seven major subfamilies of *P. massoniana* NACs are highlighted in different colored shades. Bootstrap values > 95% are shown on the corresponding branches.

Phylogenetic analysis was performed to investigate the evolutionary relationships and classifications of the *P. massoniana* NAC family proteins. A maximum likelihood phylogenetic tree was constructed using 138 previously characterized *A*. *thaliana* NAC family genes as reference sequences, together with the 50 identified genes from this study (Figure 2). According to the established classification system for Arabidopsis NAC gene families, the 50 *P. massoniana* NAC genes could be classified into 7 distinct subfamilies: ONAC003 (8 genes), NAP (7 genes), PmNAC (6 genes), ONAC022 (14 genes), NAM (2 genes), OsNAC7 (2 genes), and ANAC011 (11 genes). Importantly, no *P. massoniana* NAC genes were assigned to other Arabidopsis NAC subfamilies. Of particular interest, the PmNAC subfamily appears to be unique to *P. massoniana*, indicating potential species-specific differentiation during evolution. This distribution pattern not only reflects the evolutionary diversity of *P. massoniana* NAC genes but also suggests that their specific differentiation might confer the capacity to participate in broader regulatory networks.

### 2.4. Conserved Motif Analysis of P. massoniana NAC Family Proteins

**Figure 3:**
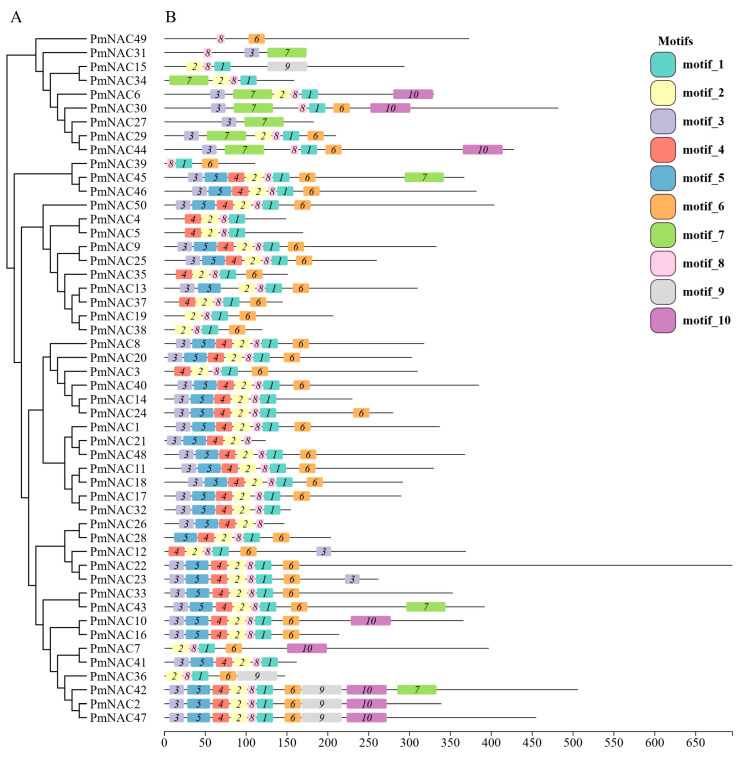
Gene structure and motif analysis of *PmNAC* genes in *P*. *massoniana.* (**A**) The phylogenetic relationships among 50 NAC genes in *P. massoniana* were inferred using a Neighbor-Joining (NJ) tree. (**B**) Conserved protein motifs within the *PmNAC* genes are depicted as color-coded boxes, with each color representing a distinct motif.

To investigate the conserved structural architecture of the 50 PmNAC family proteins, a motif analysis was conducted using the MEME suite which identified 10 conserved motifs, designated Motifs 1–10 (Figure 3). The analysis revealed that a set of motifs constitutes the core NAM domain, which is essential for the function of most PmNAC proteins. Specifically, Motifs 1, 2, and 8 were the most prevalent, found in 45, 43, and 47 of the 50 proteins, respectively. Among these, Motif 8 located in the D subdomain contains the nuclear localization signal (NLS) key sequence (KKA). Motifs 3, 4, 5, and 6 were also highly conserved, appearing in 30 to 36 members of the family. In contrast, Motifs 7, 9, and 10 were found exclusively in specific subgroups of PmNAC proteins (Figure 3), suggesting potential roles in functional specialization. Overall, the conserved nature of Motifs 1–6 and 8 underscores their fundamental role within the *P. massoniana* NAC family. Conversely, the variable distribution of the other motifs suggests that different PmNAC proteins have likely undergone functional divergence, enabling them to perform distinct biological functions in response to environmental stimuli.

### 2.5. Three-Dimensional Structure Model of PmNAC Protein

To elucidate potential functional divergences among PmNAC proteins, their three-dimensional (3D) structures were predicted and analyzed. Homology modeling was performed for representative PmNAC proteins from each subfamily using the SWISS-MODEL platform. Structural comparison revealed that the overall 3D architecture of PmNAC proteins varied considerably across different subfamilies. In contrast, proteins within the same subfamily exhibited a highly conserved spatial conformation, reflecting their close evolutionary relationships and likely shared functionalities (Supplementary Figure S1).

### 2.6. Tissue-Specific Expression Profiles of PmNAC Family Genes

**Figure 4:**
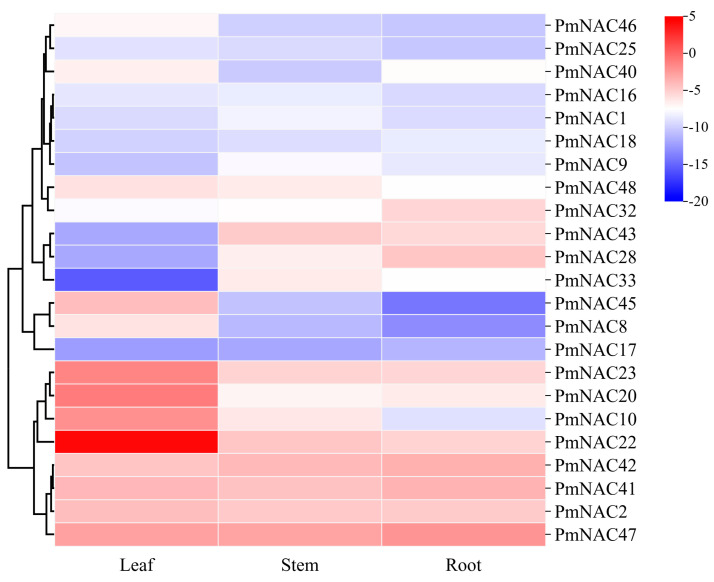
Hierarchical clustering analysis of tissue-specific expression patterns of 23 *PmNAC* genes. Heatmap visualization of qRT-PCR normalized expression levels of *PmNAC* genes in root, stem, and leaf tissues. Heatmap of gene expression normalized to *PmCYP* (internal control) using the ΔCt method. Hierarchical clustering dendrogram based on Euclidean distance and average linkage. Color scale indicates relative expression levels (blue: low; red: high).

To gain insights into the potential roles of the *PmNAC* gene family in plant development, the expression profiles of 23 representative genes, selected based on their phylogenetic distribution, were characterized across various tissues (roots, stems, and leaves) (Figure 4). The analysis revealed distinct tissue-preferential expression patterns among the selected genes. Specifically, a subset of eleven genes (e.g., *PmNAC2*, *PmNAC20*, *PmNAC40*) was predominantly expressed in leaves. Another group of six genes, including *PmNAC1* and *PmNAC9*, exhibited the highest transcript levels in stems. Conversely, six genes (e.g., *PmNAC17*, *PmNAC42*) showed preferential expression in roots (Figure 4). These distinct tissue-specific expression patterns strongly suggest that members of the PmNAC family have likely undergone functional diversification, with individual genes performing specialized regulatory functions pertinent to the development and physiology of specific organs.

### 2.7. Isolation and Identification of Nematodes and Inoculation of B. xylophilus on P. massoniana

**Figure 5:**
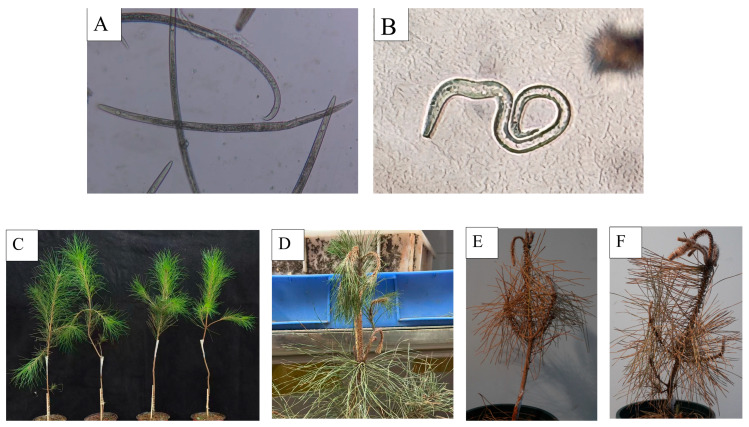
Morphological figures of *B*. *xylophilus* and changes in *P*. *massoniana* after inoculation with *B*. *xylophilus*. (**A**) Female. (**B**) Male. (**C**) The healthy *P. massoniana* plants were inoculated by *B. xylophilus*. (**D**) Two weeks after inoculation with *B. xylophilus*, the top of *P. massoniana* plants exhibited bending and wilting. (**E**,**F**) Four weeks after inoculation with *B. xylophilus*, the entire *P. massoniana* plant turned yellow and subsequently withered and died.

After isolating nematode samples from diseased plant materials, species identification was conducted by observing morphological characteristics. Under microscopic examination (Figure 5A), the nematodes exhibited an open “C”-shaped body posture after heat-killing, with a subconical tail that was blunt and rounded at the end, a single ovary, and a vulva covered by a posteriorly extended vulval flap. These features were consistent with those of female *B. xylophilus*. As shown in (Figure 5B), the heat-killed nematodes displayed a “J”-shaped body posture, with a sharp and slender tail tip, a curved abdomen, and a distal end of the spicule that was swollen and disk-shaped—characteristics consistent with male *B. xylophilus*.

Subsequently, the *B. xylophilus* nematodes were inoculated onto healthy *P. massoniana* plants (Figure 5C). One week after inoculation, the plants appeared normal, but resin secretion was reduced compared to the control group. Two weeks post-inoculation, wilting and bending at the apex of the plants were observed (Figure 5D). Four weeks after inoculation, the needles of the entire plant turned yellowish-brown, and the whole plant withered and died (Figure 5E,F). These results indicated that this *B. xylophilus* strain could induce pine wilt disease in *P. massoniana.*

### 2.8. Response of PmNAC Family Genes to Nematode Infection

**Figure 6:**
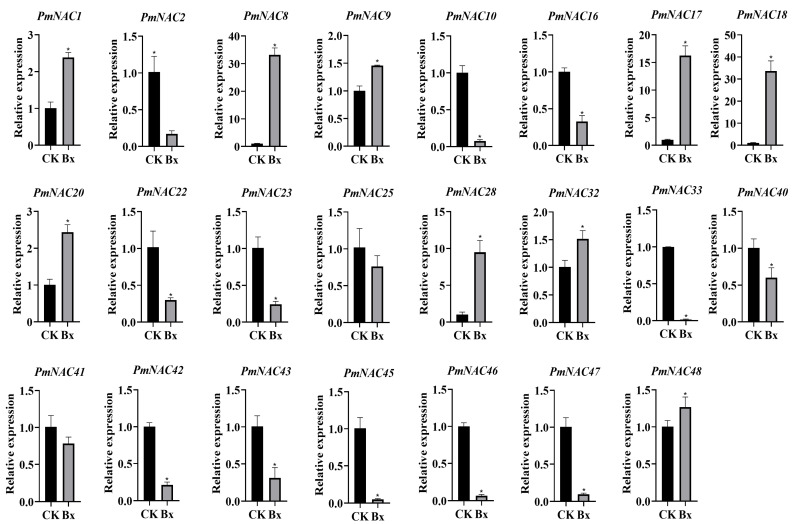
Differential expression of *PmNAC* genes in *P*. *massoniana* in response to *B*. *xylophilus* infection. The transcriptional response of 23 *PmNAC* genes to pine wood nematode (*B. xylophilus*) infection was investigated using RT-qPCR. Control samples (Control) were collected from mock-inoculated seedlings (treated with sterile water), while infected samples (BX) were collected from seedlings inoculated with nematodes. For each group, tissues from multiple whole seedlings were pooled. Relative gene expression was calculated using the 2^−∆∆Ct^ method. Data are presented as the mean ± standard deviation (SD) of three biological replicates. Student’s *t*-test was used to determine statistical significance between the infected and control groups. Asterisks denote significant differences (*, *p* < 0.05).

To elucidate the regulatory roles of *P. massoniana* NAC genes in pine wilt disease resistance, quantitative RT-PCR analysis was performed at 24 h post-inoculation with *B*. *xylophilus* (Figure 6). Notably, nine genes (*PmNAC1*, *PmNAC8*, *PmNAC9*, *PmNAC17*, *PmNAC18*, *PmNAC20*, *PmNAC28*, *PmNAC32*, and *PmNAC48*) exhibited >2-fold significant up-regulation (*p* < 0.05). In contrast, fourteen genes (*PmNAC2*, *PmNAC10*, *PmNAC16*, *PmNAC22*, *PmNAC23*, *PmNAC25*, *PmNAC33*, *PmNAC40*, *PmNAC41*, *PmNAC42*, *PmNAC43, PmNAC45, PmNAC46*, and *PmNAC47*) showed marked down-regulation (*p* < 0.05). These differential expression patterns demonstrate distinct regulatory functions of *PmNAC* genes in plant defense mechanisms. The suppressed genes may correlate with pathogen-induced growth suppression and vascular system dysfunction, characteristic symptoms of pine wilt disease.

### 2.9. Response of PmNAC Genes to Exogenous MeJA and SA

**Figure 7:**
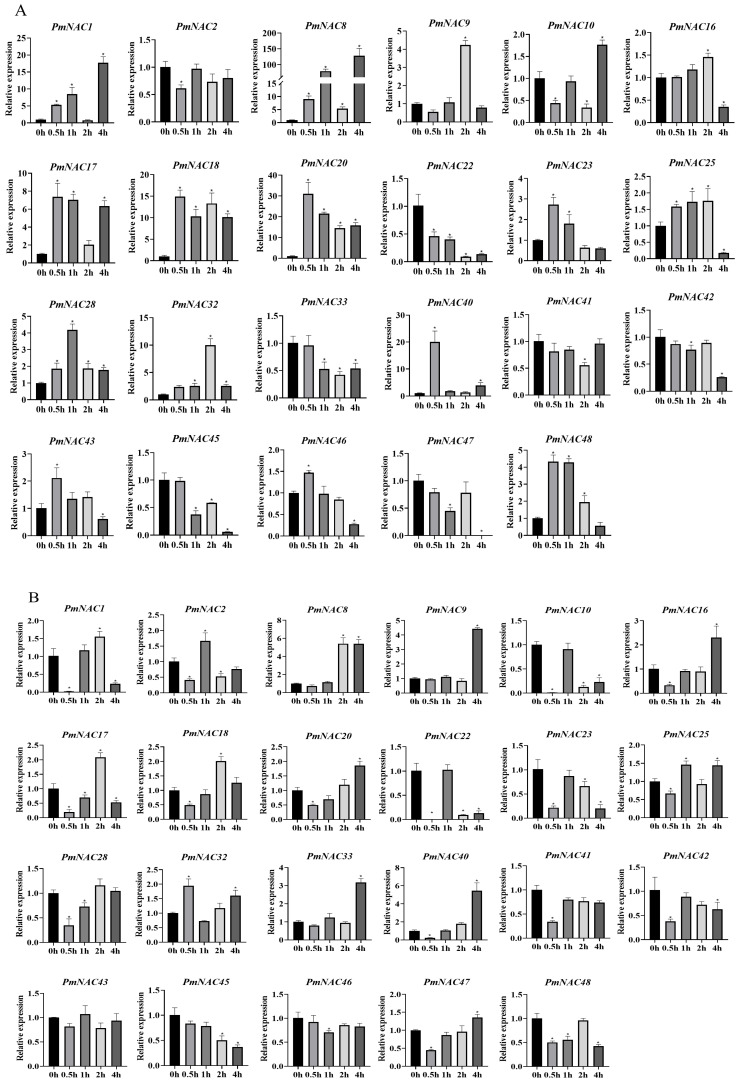
Expression analysis of the *PmNAC* gene family in *P*. *massoniana* in response to phytohormone treatments. The relative expression levels of 23 *PmNAC* genes were analyzed in *P. massoniana* seedlings (or specify the tissue, e.g., needles) after 4 h of treatment. (**A**) Plants treated with 100 μM MeJA. (**B**) Plants treated with 5 mM SA. A mock treatment (e.g., with the solvent used to dissolve the hormones) served as the control group for both experiments. Gene expression was quantified using the 2^−ΔΔCt^ method. Data are presented as the mean ± SD from three biological replicates. Asterisks indicate statistically significant differences compared to the respective mock-treated controls (* *p* < 0.05), as determined by Student’s *t*-test.

JA and SA, two pivotal phytohormones coordinating plant growth and stress adaptation, were employed to evaluate hormone-responsive expression profiles of *PmNAC* genes (Figure 7). Following 100 μM MeJA treatment for 4 h post-infiltration (hpi), 16 genes (e.g., *PmNAC1*) exhibited significant induction, with *PmNAC8* displaying the most dramatic 130.3-fold up-regulation. Conversely, 6 genes (including *PmNAC22*) showed repression, while *PmNAC2* remained non-responsive (Figure 7A). Parallel analysis under 50 μM SA treatment revealed 14 up-regulated genes, where *PmNAC8* again demonstrated 5.2-fold induction. A total of 7 genes (e.g., *PmNAC10*) were suppressed, with *PmNAC43* and *PmNAC46* showing stable expression (<1.5-fold variation) (Figure 7B). These dose-dependent responses suggest *PmNAC* genes functionally integrate into both MeJA-mediated biotic stress signaling and SA-associated systemic acquired resistance pathways.

### 2.10. Auto-Activation Activity of PmNAC8

**Figure 8:**
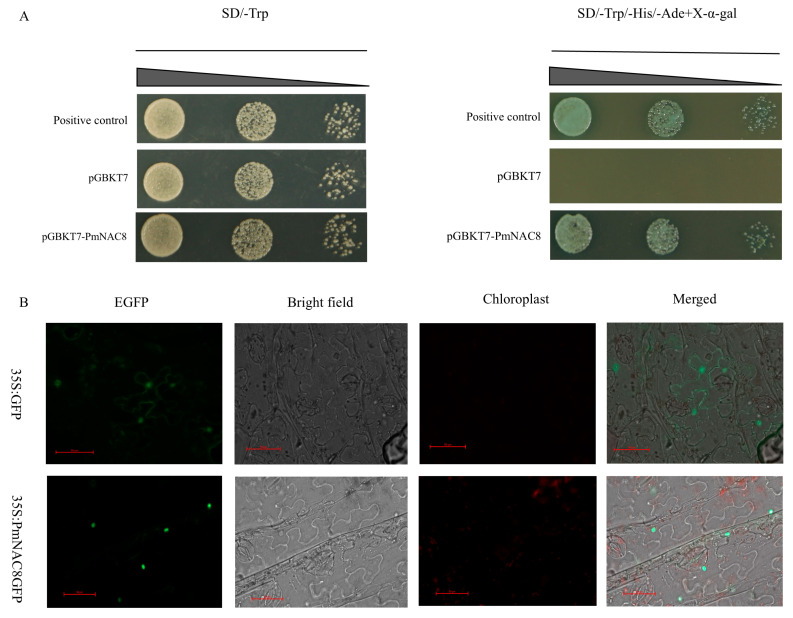
Analysis of *PmNAC8* transcription factor characteristics. (**A**) The transcriptional activation activity of *PmNAC8* was confirmed in yeast. Yeast cells transformed with a *PmNAC8* construct grew on a selective triple-deficiency medium, demonstrating its function as a transcriptional activator. (**B**) Subcellular localization analysis in *N*. *benthamiana* epidermal cells showed that the *PmNAC8*-GFP fusion protein is localized to the nucleus, consistent with its function as a transcription factor. Scale bars = 50 µm.

*PmNAC8* was identified as a prime candidate for functional analysis. It features an intact NAC domain and a canonical nuclear localization signal (NLS). Phylogenetically, it represents a distinct, lineage-specific branch divergent from known NAC subfamilies. Given that its transcription is strongly up-regulated by both nematode challenge and hormonal cues (SA, MeJA), we postulated that *PmNAC8* is a pivotal contributor to biotic stress resistance in *P. massoniana*. To test a fundamental characteristic of its predicted role as a transcription factor, we first assessed its transcriptional activation activity using a yeast one-hybrid (Y1H) system. Yeast cells transformed with *PmNAC8* successfully grew on synthetic dropout (SD)/-Trp medium, indicating the absence of cytotoxic effects. Importantly, these transformants induced reporter gene expression on selective SD/-Trp/-His/-Ade medium containing X-α-Gal and showed clear blue colony formation (Figure 8A). These results demonstrate that *PmNAC8* has intrinsic transcriptional activation activity, consistent with its predicted role as a bona fide transcription factor. Collectively, our findings suggest that *PmNAC8* has the potential to function as a nuclear regulator that orchestrates the expression of downstream target genes during the plant defense response.

### 2.11. Subcellular Localization of PmNAC8

Agrobacterium-mediated transient expression in *N*. *benthamiana* was employed to determine the subcellular localization of *PmNAC8*. The *PmNAC8-GFP* fusion protein was observed to localize exclusively in the nucleus (Figure 8B). In contrast, control leaves expressing free GFP exhibited fluorescence in both the nucleus and cytoplasm. This nuclear localization is consistent with the predicted function of *PmNAC8* as a transcription factor.

### 2.12. PmNAC8 Promotes Reactive Oxygen Species (ROS) Accumulation in Plants and Induces the Expression of Pathogenesis-Related (PR) Genes

**Figure 9:**
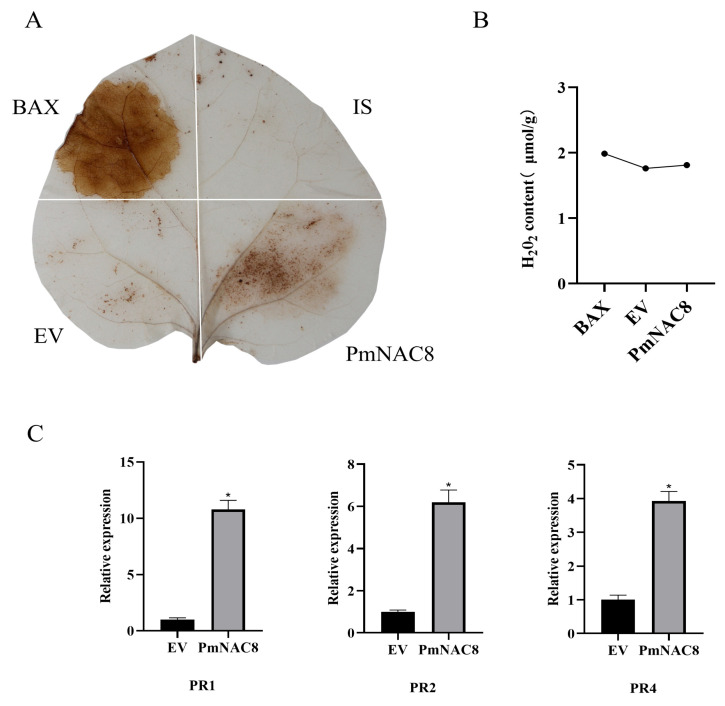
Overexpression of *PmNAC8* induces ROS accumulation and defense gene expression in *N*. *benthamiana*. (**A**) Visualization of hydrogen peroxide (H_2_O_2_) accumulation by 3,3′-diaminobenzidine (DAB) staining in *N. benthamiana* leaves at 48 h post-infiltration (hpi). Tissues were infiltrated with *Agrobacterium* carrying a *PmNAC8* expression construct or an empty vector (EV) as a control. (**B**) Quantification of H_2_O_2_ content in the infiltrated leaf tissues. (C) Relative expression levels of *pathogenesis-related* (PR) genes in the infiltrated zones, as determined by RT-qPCR. Relative gene expression was calculated using the 2^−∆∆Ct^ method. Data are presented as the mean ± standard deviation (SD) of three biological replicates. Student’s *t*-test was used to determine the statistical significance between the *PmNAC8* injection group and the empty vector group. Asterisks indicate significant differences (*, *p* < 0.05).

To elucidate the downstream defense-related functions of *PmNAC8*, we conducted a transient overexpression assay in *N*. *benthamiana* leaves via Agrobacterium-mediated infiltration. At 48 h post-infiltration (hpi), two key defense responses were evaluated: the production of reactive oxygen species (ROS) and the expression of *pathogenesis-related* (*PR*) genes. A substantial ROS burst was observed in leaf areas infiltrated with the *35S::PmNAC8* construct, as visualized by 3,3′-diaminobenzidine (DAB) staining (Figure 9A). Consistent with this result, quantitative measurement of hydrogen peroxide (H_2_O_2_) content revealed significantly higher levels in *PmNAC8*-expressing tissues (1.81 μmol/g) compared with the empty vector (EV) control (1.76 μmol/g), albeit lower than the positive control BAX (1.98 μmol/g) (Figure 9B). Furthermore, qRT-PCR analysis showed that the relative expression levels of *NbPR1* (pathogenesis-related protein 1), *NbPR2* (β-1,3-Glucanase), and *NbPR4* (chitin-binding protein) were increased by approximately 10.7-, 6.1-, and 3.9-fold, respectively, compared to the EV control (Figure 9C).

## 3. Discussion

The NAC gene family, widely distributed across the plant kingdom, comprises pivotal regulators of plant growth, development, and stress responses [24,26,27]. While extensive studies have characterized NAC families in diverse angiosperms, comprehensive analyses in gymnosperms, particularly conifers, remain relatively scarce [28]. In this study, we identified 50 putative PmNAC members from the *P. massoniana* transcriptome and classified them into 7 subfamilies. This phylogenetic grouping aligns with the established classification of NAC families in other model plants, suggesting a conserved evolutionary history [19,28].

Our analyses revealed several conserved features within the PmNAC family. For instance, physicochemical predictions indicated that most PmNACs are basic proteins, a common characteristic of transcription factors that facilitates binding to negatively charged DNA [29]. Furthermore, the distribution of conserved motifs largely corresponded with the phylogenetic groupings, providing additional support for our classification. The highly conserved N-terminal NAC domain, containing the characteristic A to E subdomains, was identified in 23 PmNAC members, underscoring their structural conservation with well-characterized *Arabidopsis* NACs. Taken together these results demonstrate that the *P. massoniana* NAC family exhibits both high structural and evolutionary conservation alongside lineage-specific characteristics, which may contribute to its adaptation to diverse environmental stimuli.

Phytohormones act as essential signaling molecules regulating plant development and stress responses, particularly under biotic stress. Upon pathogen or pest recognition, plant cells activate pattern-triggered immunity (PTI) or effector-triggered immunity (ETI), resulting in the accumulation of hormones including SA and JA [30,31]. In susceptible plants, MeJA and SA levels increase significantly following nematode infection [5]. In our study, exogenous MeJA treatment rapidly up-regulated 14 *PmNAC* genes (including *PmNAC1*), whereas SA treatment caused transient up-regulation followed by subsequent down-regulation in 12 genes. The quicker response to MeJA indicates that *PmNAC* genes are primarily involved in JA signaling during exogenous stress [32]. Under *B. xylophilus* infection, nematode treatment led to a 1.2- to 33-fold up-regulation of nine *PmNAC* genes, with *PmNAC8* exhibiting the strongest induction (33-fold), suggesting their involvement in activating disease resistance mechanisms. In contrast, fourteen genes (e.g., *PmNAC2*) were down-regulated 1.2- to 66-fold, potentially due to nematode-induced suppression or a controlled defense strategy to avoid excessive early response and support long-term resistance [33]. Notably, although *PmNAC8* is highly expressed in needles, it showed marked induction by stem nematode infestation, implying a role in long-distance signaling. This nuclear-localized protein demonstrates transcriptional autoactivation activity and is highly responsive to SA, JA, and nematode challenge. Collectively, our findings suggest that *PmNAC8* likely activates downstream JA signaling cascades in the nucleus to enhance resistance against nematode invasion.

The production of reactive oxygen species (ROS), often termed the “oxidative burst,” is a hallmark of plant immunity, acting as a critical signaling molecule and a direct antimicrobial agent following pathogen recognition [15]. The magnitude and kinetics of this ROS production, frequently generated by NADPH oxidases like RBOHs, are often correlated with the level of plant resistance and are integral to both PAMP-triggered (PTI) and effector-triggered immunity (ETI) [34]. Given the essential role of ROS in plant defense, we investigated whether *PmNAC8* could function as an upstream regulator of this pathway. Our heterologous expression experiments in *N. benthamiana* provided compelling evidence. The transient overexpression of *PmNAC8* was sufficient to induce a significant accumulation of ROS, a response characteristic of the hypersensitive response (HR). This finding was further corroborated by the concomitant and significant up-regulation of downstream pathogenesis-related (*PR*) genes. Collectively, these results demonstrate that *PmNAC8* is not merely a stress-responsive gene but a potent, functional activator of core defense mechanisms. This capability strongly suggests that the robust induction of *PmNAC8* observed during *B. xylophilus* infection in *P. massoniana* serves as a key regulatory event to initiate a defensive cascade, thereby contributing to nematode resistance.

In conclusion, the NAC gene family in *P. massoniana* displays evolutionary conservation while exhibiting lineage-specific diversification, likely facilitating adaptation to both biotic and abiotic stresses. This study identifies phylogenetically divergent members, such as *PmNAC8*, as central to specialized defense mechanisms. We demonstrate that *PmNAC8* acts as a nuclear-localized transcription factor and a crucial positive regulator of pine immunity. Specifically, *PmNAC8* integrates defense signaling and induces ROS accumulation, coordinating downstream immune responses against the pine wood nematode *B. xylophilus*. These results not only clarify the molecular basis of *P. massoniana* resistance, but also establish *PmNAC8* as a promising candidate for genetic improvement of pine wilt disease (PWD) resistance through molecular breeding and genetic engineering.

## 4. Materials and Methods

### 4.1. Physicochemical Property Analysis of the NAC Family in P. massoniana

The open reading frames (ORFs) and protein sequences of the NAC family in *P. massoniana* were obtained from the Plant Genome Database (accession code: t88731.g001; available at: https://plantgarden.jp; accessed on 17 December 2023). Domain architecture verification was performed using SMART (v9.0; http://smart.embl.de; accessed on 27 January 2024) and the Conserved Domain Database (CDD v3.20; https://www.ncbi.nlm.nih.gov/cdd; accessed on 27 January 2024) [35], with subsequent analyses confirming the presence of NAM domains in all 50 candidate proteins. Key physicochemical properties (including molecular weight, isoelectric point, instability index, and grand average of hydropathicity) were determined using the ExPASy ProtParam tool (https://web.expasy.org/protparam; accessed on 29 January 2024) [36]. Protein secondary structure composition (α-helices, β-strands, and random coils) was predicted through the SOPMA algorithm (SOPMA Secondary Structure Prediction, https://npsa-prabi.ibcp.fr; accessed on 30 January 2024).

### 4.2. Sequence Alignment and Phylogenetic Analysis

To investigate the functional divergence and evolutionary patterns of NAC family proteins, reference sequences from *A. thaliana* (138 NAC members; retrieved from Plant Transcription Factor Database v5.0 (PlantTFDB, PMID: 33219652; http://planttfdb.cbi.pku.edu.cn; accessed on 27 January 2024) were aligned with *P. massoniana* homologs. Multiple sequence alignments were generated via the MUSCLE algorithm (v3.8.31) implemented in MEGA version 12.0 (Molecular Evolutionary Genetics Analysis) under default parameters, with subsequent curation of domain boundaries. The optimal substitution model (LG + G) was selected through Bayesian Information Criterion (BIC) in MEGA, and a maximum likelihood phylogenetic tree was reconstructed using 1000 bootstrap replicates for node support. Final clade annotations were performed according to established NAC subgroup nomenclature and visualized through ChiPlot v3.0 (ChiPlot Scientific Online Tools; https://www.chiplot.online; accessed on 20 February 2024).

### 4.3. Conserved Motif Prediction and Subdomain Analysis

The amino acid sequences of all identified PmNAC proteins were subjected to multiple sequence alignment using DNAMAN to examine for the presence of the five characteristic NAC subdomains (A–E). Conserved motifs within the PmNAC protein sequences were identified using the MEME Suite (v5.5.0) [37]. The analysis was conducted with the following parameters: a maximum of 10 motifs to be discovered, and an individual motif width ranging from 6 to 60 amino acids. Subsequently, the identified motif architecture was visualized using the ChiPlot online tool [38].

### 4.4. Tertiary Structure Modeling

Three-dimensional structures of NAC family proteins were modeled using the SWISS-MODEL server (https://swissmodel.expasy.org; accessed on 25 February 2024), with default template parameters and sequence coverage criteria [39].

### 4.5. Healthy Plant Materials

Healthy *P. massoniana* saplings were cultivated at the Masson Pine Specialty Forest Farm (Hunan Forestry Bureau). The farm utilizes greenhouse structures enclosed with light-permeable, aerated fine-mesh netting to prevent the entry of external species (e.g., *Monochamus alternatus*, vector of *B. xylophilus*). Healthy *N. benthamiana* plants were grown in a controlled greenhouse at 26 °C with a 12 h photoperiod. Plants were protected from aphid infestation and used after four weeks of cultivation.

### 4.6. Isolation and Identification of PWN

Samples were collected from wilted *P. massoniana* trunks at the Masson Pine Breeding Station (Hunan Academy of Forestry). Xylem fragments (50 g each) from upper, middle, and lower trunk sections were processed using the Baermann funnel technique of being soaked for 24 h [40], followed by nematode isolation. Specimens were examined under stereomicroscopy and light microscopy. Females: Open C-shape Tail, Subconical with blunt terminus; minority exhibit minute mucron (<2 μm) Reproductive system, Single anterior ovary; oocytes linearly arranged Vulva: Positioned at ~73% body length, covered by prominent anterior vulval flap. Males: J-shaped curvature Tail: Sharply pointed; ventrally curved with oval bursa Spicules: Paired, arched, prominent rostrum; distally expanded (disk-shaped). Larvae: Anterior region: Adult-like morphology; Posterior region: Granular intestinal contents causing darkened appearance tail, subconical, mucron absent or reduced (Figure 5) [41,42]. When morphological identification is inconclusive, specific primers and probes designed based on the rDNA-ITS2 region of *B. xylophilus* are used to perform real-time fluorescent quantitative PCR (qRT-PCR) on the nematodes, thereby determining whether they belong to *B. xylophilus* [43].

### 4.7. Inoculation of the PWN and Sample Preparation

a. *B. xylophilus* nematodes isolated via Baermann funnel were examined microscopically for species identification and viability confirmation. High-viability nematodes were selected for inoculation. b. A “Y”-shaped wound (depth: xylem-exposing) was created on the main stem of healthy two-year-old *P. massoniana* saplings (~10 cm below branches) using sterile surgical scalpels. c. A 200 μL nematode suspension (~2000 PWNs) was applied to the wound site, sealed with breathable medical tape. Parafilm was wrapped below the wound to prevent leakage. Control groups were treated with sterile water. Following inoculation, all *P. massoniana* seedlings were maintained in a greenhouse at 26 °C under a 12 h photoperiod until symptom assessment. d. Plants were monitored for weeks until apical bending and chlorosis appeared (Figure 5). Whole-stem xylem sections were processed using the Baermann protocol to confirm successful inoculation. Progressive whole-plant chlorosis and mortality—matching field-infected *P. massoniana* symptoms—confirmed PWN pathogenicity. e. Roots, stems, and leaves from PWN-infected plants were pooled for total RNA extraction.

### 4.8. Verification of Pathogenicity of PWN

We collected *P. massoniana* plants infected with *B. xylophilus* from a forest farm. Nematodes were isolated using the Baermann funnel method, and their morphology was observed under a microscope before inoculation using the aforementioned method. The inoculated plants were maintained at 26 °C under a photoperiod of more than 12 h of light. Two weeks after inoculation, disease severity was evaluated using the grading guidelines for PWN disease established by the International Union of Forest Research Organizations (IUFRO). A 0–4 scale was used: 0: Asymptomatic; 1: Apical wilting; 2: >50% needle chlorosis + stem bending; 3: >75% needle desiccation + branch dieback; 4: Plant death. Subsequently, *B. xylophilus* nematodes were isolated again from the diseased plants using the Baermann funnel method, and their morphology was observed under a microscope to confirm consistency with the original inoculum type. Real-time fluorescent PCR with specific primers was then used to identify these nematodes as *B. xylophilus*. Thereafter, the isolated nematodes were inoculated into healthy *P. massoniana* plants, which subsequently exhibited consistent disease symptoms, thus confirming that the yellowing and wilting of *P. massoniana* were caused by this species of *B. xylophilus* [44]. Disease Index% (DI%) is used to evaluate the severity of the disease. The DI% was calculated as follows:

$$DI\%=\frac{\Sigma xy}{nz}\times 100\%$$

where *x* is the value of severity grade, *y* is the number of plants in that grade, *n* is the value of the highest disease severity gradient (*n* = 4), and *z* is the total number of plants [45].

### 4.9. SA and MeJA Processing and Material Preparation

To investigate the response patterns of *P. massoniana* NAC family genes to exogenous SA and MeJA and to explore whether the NAC family in *P. massoniana* participates in SA- and JA-mediated pathways in response to biotic invasion, we conducted the following experiments. We prepared a 5 mM SA solution and a 100 μM MeJA solution [46]. Two-year-old healthy *P. massoniana* plants cultivated in the forest farm were selected, and the entire plant (including all leaves and stems) was sprayed uniformly with the solutions using a spray bottle. Immediately after spraying, the plants were wrapped with transparent plastic wrap to prevent the volatilization of the two hormones, and three treatment groups were set up. At 0 h post-treatment (hpt), 5 g of leaves and partial branch stems from untreated plants were collected. Due to the rapid response of plants to hormones, samples (5 g of leaves and branch stems from different positions of the plant) were collected at 0.5, 1, 2, and 4 hpt. During each sampling, only one small hole was used to collect samples, and the sampling site was immediately sealed after collection to avoid hormone volatilization.

### 4.10. RNA Isolation and qRT-PCR

Total RNA was extracted using an RNAprep Pure Plant Tissue Kit (Tiangen Biotech, Beijing, China) according to the manufacturer’s instructions, quantified using a NanoDrop 2000 spectrophotometer (Thermo Fisher Scientific, Waltham, MA, USA), and assessed by agarose gel electrophoresis. First-strand cDNA was synthesized using a HP All-in-One qRT MasterMix II (Yungeng Bio, Kunming, China). Quantitative real-time PCR (qRT-PCR) was performed for 17 selected genes to analyze their expression patterns across different tissues (root, stem, and leaf), during nematode infection, and under hormone treatments (Table S2). The resulting expression profiles suggested roles for NAC genes in growth, development, and stress responses. qRT-PCR assays were carried out under the following cycling conditions: initial denaturation at 95 °C for 30 s, followed by 40 cycles of 95 °C for 10 s and 60 °C for 30 s. Each 20 μL reaction mixture contained 1 μL of cDNA template, 10 μL of 2× Taq Pro Universal SYBR qPCR Master Mix (Vazyme, Nanjing, China), 0.4 μL of each primer (10 μM), and 8.2 μL of ddH_2_O. PCR amplifications were conducted using a Bio-Rad real-time PCR detection system (Foster City, CA, USA), with three biological replicates for each sample. CYP was used as the reference gene for normalization [47].

### 4.11. Yeast Self-Activation Assay

To evaluate the transcriptional activation activity of the *P. massoniana* NAC family, one representative gene (*PmNAC8* from the PmNAC subfamily) was selected based on domain alignment, phylogenetic analysis, and its differential expression in response to nematode infection. The full-length coding sequences (CDSs) of *PmNAC8* were amplified and cloned into the pGBKT7 vector, generating fusions with the GAL4 DNA-binding domain. The recombinant plasmids were introduced into the yeast strain AH109 by heat shock transformation. Transformants were screened on selective media (SD/-Trp and SD/-Trp/-His/-Ade supplemented with X-α-gal) at 28 °C for 2–4 days. Positive colonies were further confirmed for plasmid integration by colony PCR and re-streaking assays.

### 4.12. Subcellular Localization Analysis

To determine the subcellular localization of PmNAC proteins, the online tools Plant-mPLoc (http://www.csbio.sjtu.edu.cn/bioinf/plant-multi/, (accessed on 28 February 2025)) [48] and WoLF PSORT (https://psort.hgc.jp/, (accessed on 28 February 2025)) [49] were employed for prediction. Both algorithms indicated that *PmNAC8* is localized to the nucleus. The full-length *PmNAC8* coding sequence was fused in-frame with GFP and cloned into an *Agrobacterium* expression vector. The resulting recombinant plasmid was introduced into *Agrobacterium tumefaciens* strain GV3101 using a freeze–thaw transformation method (1 μg plasmid added to competent cells, followed by incubation on ice, rapid freezing in liquid nitrogen, and recovery in LB medium at 28 °C). Positive transformants were selected on LB plates containing kanamycin and rifampicin and subsequently verified by colony PCR. The *PmNAC8–GFP* fusion construct was transiently expressed in *N. benthamiana* leaves. Fluorescence localization was examined 48 h post-infiltration under a confocal laser scanning microscope and compared with empty vector controls.

### 4.13. Transient Overexpression of PmNAC8 in N. benthamiana

The coding sequence of *PmNAC8* was cloned into the pCAMBIA1301 vector under the control of the Cauliflower mosaic virus (CaMV) 35S promoter to generate the *35S::PmNAC8* construct. The construct, along with the empty vector (EV) pCAMBIA1301 (negative control) and a construct expressing the pro-apoptotic protein BAX (positive control), was transformed into the *Agrobacterium tumefaciens* strain GV3101. For transient expression, *Agrobacterium* cultures were grown, harvested, and resuspended in an infiltration buffer (10 mM MgCl_2_, 10 mM MES, pH 5.6, 200 μM acetosyringone) to a final optical density (OD_600_) of 0.8. The bacterial suspensions were then infiltrated into the abaxial side of leaves of 4-week-old *N. benthamiana* plants. Infiltrated plants were maintained in darkness for 8 h and subsequently grown under a 16 h light/8 h dark photoperiod at 25 °C. At 48 h post-infiltration (hpi), the infiltrated leaf zones were photographed to document phenotypes. For subsequent analyses, leaf disks were harvested from the infiltrated areas.

### 4.14. Detection of Reactive Oxygen Species (ROS)

To visualize hydrogen peroxide (H_2_O_2_) accumulation, infiltrated leaf samples were subjected to 3,3′-diaminobenzidine (DAB) staining. Leaf disks were immersed in a 1 mg/mL DAB solution (pH 5.8) and incubated in the dark for 8 h. Following incubation, chlorophyll was removed by boiling in 95% ethanol prior to imaging. For quantitative measurement, H_2_O_2_ content was determined using a Hydrogen Peroxide Assay Kit (Solarbio, Beijing, China) following the manufacturer’s instructions.

### 4.15. Statistical Analysis

The relative expression levels were calculated using the 2^−ΔΔCt^ method, with results presented as mean ± standard error and with statistical significance indicated by (* *p* ≤ 0.05) as determined by Student’s *t*-test. Heatmap of gene expression was normalized to *PmCYP* (internal control) using the ΔCt method. A hierarchical clustering dendrogram based on Euclidean distance and average linkage was created. The color scale indicates relative expression levels (blue: low; red: high).

## 5. Conclusions

Our study reveals the pivotal role of the NAC transcription factor family in the defense of *P. massoniana* against pine wilt disease (PWD). While 50 *PmNAC* genes exhibit both evolutionary conservation and conifer-specific diversification, we identified a key stress-responsive member, *PmNAC8*, as a central regulator of immunity. Functional analyses confirmed that *PmNAC8* is a nuclear-localized transcriptional activator that triggers robust defense responses, including ROS accumulation. These findings establish *PmNAC8* as a positive regulator in the SA/JA-mediated signaling pathways against *B. xylophilus*. This work not only clarifies a critical molecular mechanism of pine defense but also provides a prime genetic target for developing PWD-resistant varieties.

## Data Availability

Data are contained within the article.

## Supplementary Materials

The following supporting information can be downloaded at: https://www.mdpi.com/article/10.3390/plants14152399/s1, Table S1. Summary of Physicochemical Characteristics for 50 NAC Transcription Factor Proteins. Table S2. Sequence-Specific Primers for qRT-PCR Analysis of Target Genes. Figure S1. Three-Dimensional Structure Model of 50 PmNAC Protein.
