# Supplementary material for: Identification and Characterization of NAC Transcription Factors Involved in Pine Wilt Nematode Resistance in Pinus massoniana
Source: Plants (Basel). 2025 Aug 3;14(15):2399. doi: 10.3390/plants14152399 (PMC12349346; doi:10.3390/plants14152399)
Supplement: Supplementary file 1 [file plants-14-02399-s001.zip › Table S1.pdf]

Supplemental Table S1. Summary of Physicochemical Characteristics for 50 NAC Transcription Factor Proteins

Gene name	Amino Acid Number	Molecular Weight (kDa)	Theoretical pI	Instability Index	Hydropathicity	Subcellular Localization	Alpha helix		Extended strand		Beta turn		Random coil	
							The state	Ratio (%)	The state	Ratio (%)	The state	Ratio (%)	The state	Ratio (%)
PmNAC1	337	38.3	8.1	44.19	-0.691	Nuclear	19	5.64	40	11.87	0	0	278	82.49
PmNAC2	339	38.5	5.0	41.70	-0.724	Nuclear	29	8.55	37	10.91	0	0	273	80.53
PmNAC3	310	35.2	8.5	41.36	-0.745	Nuclear	15	4.84	26	8.39	0	0	269	86.77
PmNAC4	149	16.9	8.3	29.70	-0.193	Nuclear	18	12.08	37	24.83	0	0	94	63.09
PmNAC5	170	19.7	9.3	46.03	-0.566	Nuclear	12	7.06	32	18.82	0	0	126	74.12
PmNAC6	330	38.2	5.4	51.82	-0.783	Nuclear	44	13.33	44	13.33	0	0	242	73.33
PmNAC7	397	45.8	4.7	52.58	-0.852	Nuclear	36	9.07	20	5.04	0	0	341	85.89
PmNAC8	318	36.7	8.7	41.79	-0.740	Nuclear	17	5.35	35	11.01	0	0	266	83.65
PmNAC9	333	38.2	4.8	42.24	-0.579	Nuclear	33	9.91	34	10.21	0	0	266	79.88
PmNAC10	366	41.7	5.1	42.44	-0.642	Nuclear	26	7.10	37	10.11	0	0	303	82.79
PmNAC11	330	37.5	6.9	61.94	-0.536	Nuclear	29	8.79	36	10.91	0	0	265	80.30
PmNAC12	369	42.8	4.4	43.47	-0.682	Nuclear	30	8.13	28	7.59	0	0	311	84.28
PmNAC13	310	36.8	6.3	44.86	-0.571	Nuclear	19	6.13	43	13.87	0	0	248	80.00
PmNAC14	230	26.8	9.8	41.36	-0.313	Nuclear	27	11.74	39	16.96	0	0	164	71.30
PmNAC15	294	33.4	5.1	54.31	-0.969	Nuclear	23	7.82	28	9.52	0	0	243	82.65
PmNAC16	214	24.3	5.9	47.72	-0.894	Nuclear	24	11.21	32	14.95	0	0	158	73.83
PmNAC17	290	33.4	9.2	72.00	-0.465	Nuclear	30	10.34	35	12.07	0	0	225	77.59
PmNAC18	292	33.2	7.8	45.00	-0.466	Nuclear	40	13.70	40	13.70	0	0	212	72.60
PmNAC19	207	23.1	9.0	47.83	-0.647	Nuclear	12	5.80	27	13.04	0	0	168	81.16
PmNAC20	303	34.7	8.2	47.30	-0.767	Nuclear	16	5.28	37	12.21	0	0	250	82.51
PmNAC21	124	14.3	9.7	33.83	-0.715	Nuclear	9	7.26	32	25.81	0	0	83	66.94
PmNAC22	695	77.4	4.9	41.07	-0.484	Nuclear	83	11.94	41	5.90	0	0	571	82.16
PmNAC23	262	29.8	6.5	40.78	-0.783	Nuclear	19	7.25	40	15.27	0	0	203	77.48
PmNAC24	280	31.9	9.2	30.46	-0.049	Nuclear	55	19.64	48	17.14	0	0	177	63.21
PmNAC25	260	30.1	8.6	38.63	-0.758	Nuclear	24	9.23	35	13.46	0	0	201	77.31
PmNAC26	147	16.8	8.6	41.47	-0.701	Nuclear	30	20.41	24	16.33	0	0	93	63.27
PmNAC27	183	20.5	9.0	36.92	-0.879	Nuclear	26	14.21	32	17.49	0	0	125	68.31

Supplemental Table S1 (cont.). Summary of Physicochemical Characteristics for 50 NAC Transcription Factor Proteins

Gene name	Amino Acid Number	Molecular Weight (kDa)	Theoretical pI	Instability Index	Hydropathicity	Subcellular Localization	Alpha helix		Extended strand		Beta turn		Random coil	
							The state	Ratio (%)	The state	Ratio (%)	The state	Ratio (%)	The state	Ratio (%)
PmNAC28	204	23.1	8.7	30.44	-0.669	Nuclear	22	10.87	36	17.65	0	0	146	71.57
PmNAC29	210	23.5	8.9	48.72	-0.954	Nuclear	25	11.9	42	20.00	0	0	143	68.1
PmNAC30	482	54.8	5.9	49.23	-0.852	Nuclear	71	14.73	46	9.54	0	0	365	75.73
PmNAC31	174	19.8	6.4	33.21	-0.595	Nuclear	28	16.09	25	14.37	0	0	121	69.54
PmNAC32	155	17.8	6.2	41.09	-0.507	Nuclear	19	12.26	34	21.94	0	0	102	65.81
PmNAC33	353	40.5	5.9	48.94	-0.863	Nuclear	38	10.76	36	10.20	0	0	279	79.04
PmNAC34	159	18.5	9.1	40.49	-0.955	Nuclear	15	9.43	38	23.90	0	0	106	66.67
PmNAC35	151	17.3	9.4	44.59	-0.761	Nuclear	10	6.62	26	17.22	0	0	115	76.16
PmNAC36	148	16.3	9.1	34.63	-0.683	Nuclear	20	13.51	17	11.49	0	0	111	75.00
PmNAC37	145	16.8	9.6	24.71	-0.787	Nuclear	10	6.9	28	19.31	0	0	107	73.79
PmNAC38	120	13.5	9.4	28.75	-0.830	Nuclear	11	9.17	20	16.67	0	0	89	74.17
PmNAC39	111	12.8	9.5	52.08	-1.279	Nuclear	6	5.41	17	15.32	0	0	88	79.28
PmNAC40	385	43.8	6.7	48.37	-0.757	Nuclear	29	7.53	35	9.09	0	0	321	83.38
PmNAC41	162	18.6	9.3	44.8	-0.900	Nuclear	28	17.28	36	22.22	0	0	98	60.49
PmNAC42	506	56.8	4.8	46.71	-0.702	Nuclear	50	9.88	36	7.11	0	0	420	83.01
PmNAC43	392	45.3	6.4	43.62	-0.827	Nuclear	31	7.91	37	9.44	0	0	324	82.65
PmNAC44	428	47.9	6.0	50.30	-0.836	Nuclear	36	8.41	44	10.28	0	0	348	81.31
PmNAC45	367	42.3	7.8	44.22	-0.884	Nuclear	39	10.63	32	8.72	0	0	296	80.65
PmNAC46	382	43.5	7.4	54.21	-0.653	Nuclear	40	10.47	37	9.69	0	0	305	79.84
PmNAC47	455	51.2	4.9	49.87	-0.654	Nuclear	36	7.91	35	7.69	0	0	384	84.4
PmNAC48	368	42.1	6.1	42.47	-0.873	Nuclear	36	9.78	38	10.33	0	0	294	79.89
PmNAC49	373	42.8	4.8	37.96	-0.871	Nuclear	24	6.43	35	9.38	0	0	314	84.18
PmNAC50	404	46.3	6.2	44.89	-0.697	Nuclear	27	6.68	35	8.66	0	0	342	84.65
