# Supplementary material for: Identification and Characterization of NAC Transcription Factors Involved in Pine Wilt Nematode Resistance in Pinus massoniana
Source: Plants (Basel). 2025 Aug 3;14(15):2399. doi: 10.3390/plants14152399 (PMC12349346; doi:10.3390/plants14152399)
Supplement: Supplementary file 1 [file plants-14-02399-s001.zip › Table S2.pdf]

Supplemental Table S2. Sequence-Specific Primers for qRT-PCR Analysis of Target Genes

Gene name	Forward Primer (5'-3')	Reverse Primer (5'-3')
PmNAC1	CCACCCCACAGATGAAGAGC	CCAGTACCCAGAAGCAGCAG
PmNAC2	CCAGGTTTCAGGTTCCATCCC	GGCTGGGCAGGAAGGATTTC
PmNAC8	CAACCGAGCTGCAGGATCTG	ACACCTCTAGGAGCCTTCCC
PmNAC9	GGGGACAGAAGACCGATTGG	CAGGTCGACTTAGCCTCCTG
PmNAC10	CGGGTTTCAGGTTCCATCCC	GCCCTATTTGTGCGTGTTCC
PmNAC16	TGGGACTTGCCTGAGAGGTC	CTCCGGTCCTTTCCTGTAGC
PmNAC17	GTGCCAGAGCTGCTTGTAGG	GGGTCGTGTTGGTAGGGATC
PmNAC18	AGTCTCCTGTTCGCCTCTCC	CTGTTCCCGTGGCTTTCCAG
PmNAC20	CCGGCGCAGATAAACCCATC	GGCACAGCACCCAATCATCC
PmNAC22	CCATCCCACTGACGAAGAGC	TGCCTGGTAAATCCCAAGGC
PmNAC23	GAGGCCGATCCATACGAGTG	CGAGTGACCTTCCAGTAACCC
PmNAC25	GGTTCCACCCTACAGAGCAG	CACCTCTCATCTCGCGGAAC
PmNAC28	CACAAACCGAGCCACAGAGG	TGGGTGCTCTCCCCTTGTAG
PmNAC32	CGGCTCTGGCTACTGGAAAG	CCTCACCGGCCTTTCCTTTG
PmNAC33	TAGGGTCCGAGGAACAGACG	CGCGGCCTTTGTAGAAGACC
PmNAC40	CCAGAGAAGGCCCTGTTTGG	AGAACCAGCAGCCCTATTGG
PmNAC41	CCCCGGGATTCAGATTTCGG	GATCCCAGGGCTCACACTTG
PmNAC42	CAGGTTCCATCCCACTGACG	CGCTGGGCAGGAAGGATTTC
PmNAC43	CTGGACGACGCCGAATATCC	GCTGGGGCAATCTGAGTCTG
PmNAC45	CCGGATGGTTCGCAATGATG	CACGGCAAAGGGAGAGTTCG
PmNAC46	GGCGCCCAAAGGAAAAAGG	CAGACCCTCCTGGCCTTTTG
PmNAC47	GCTATGCCAGACACTCCAAG	CCTCTGAGGCCACAGACAAC
PmNAC48	CCTTCTCGGAGTCGCATCAG	CCTGCTCTCCAAAGGTTGCC
PmCYP	CAAGGGTTCGTCGTTCCAC	GGCAAACTTCTCGCCGTA
NbACT	TCCTGATGGGCAAGTGATTAC	TTGTATGTGGTCTCGTGGATTC
PR1	ACAAATGGCCTAACACAGCA	TCCGGACTTCTCTCACCTCT
PR2	CAATGGAGAAGTGATGCCCG	CTACACACTCAAGGCTCGGA
PR4	TGCGTGGCATTGTTGATCAT	AGTGAAGGGCATGTAGAGCT
BX	GGCGAGAGGGCTTCG	CGCCGTTGAAACAACATCAC
BXP-TaqMan	ATTGTCGTGCGCGGCTAAACCGT	
