# Supplementary figures and images for: Identification and Characterization of NAC Transcription Factors Involved in Pine Wilt Nematode Resistance in Pinus massoniana
Source: Plants (Basel). 2025 Aug 3;14(15):2399. doi: 10.3390/plants14152399 (PMC12349346; doi:10.3390/plants14152399)

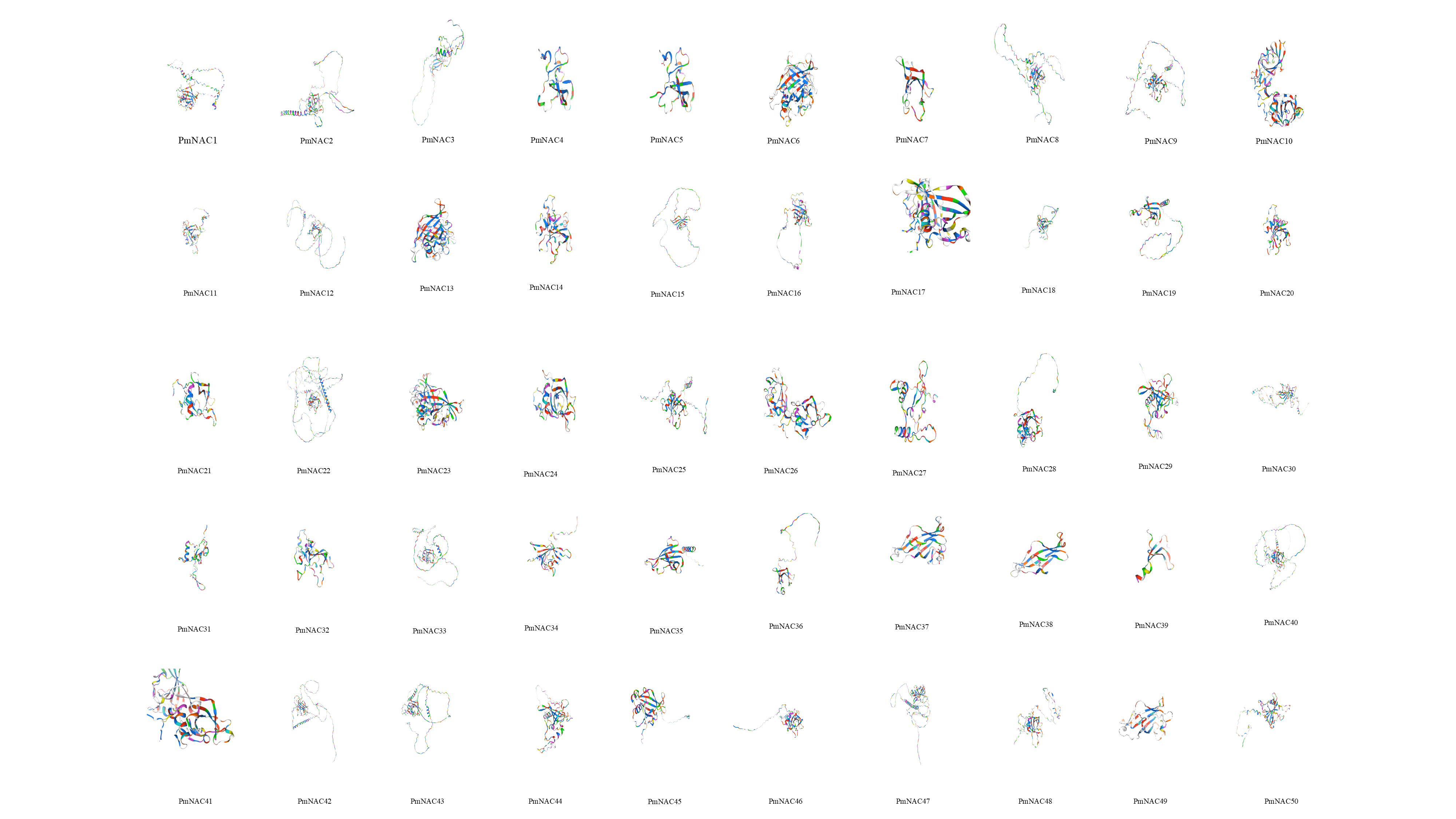

Supplement: Supplementary file 1 [file plants-14-02399-s001.zip › Supplementary Figure S1.tif]
